# Neuromodulation in Pediatric Migraine using Repetitive Neuromuscular Magnetic Stimulation: A Feasibility Study

**DOI:** 10.3390/children10111764

**Published:** 2023-10-30

**Authors:** Corinna Börner-Schröder, Magdalena Lang, Giada Urban, Erik Zaidenstadt, Jacob Staisch, Ari Hauser, Iris Hannibal, Kristina Huß, Birgit Klose, Matthias F. Lechner, Nico Sollmann, Mirjam N. Landgraf, Florian Heinen, Michaela V. Bonfert

**Affiliations:** 1 Division of Pediatric Neurology and Developmental Medicine, Department of Pediatrics, Dr. Von Hauner Children’s Hospital, LMU University Hospital, LMU Munich, 80337 Munich, Germany; corinna.boerner@med.lmu.de (C.B.-S.); iris.hannibal@med.lmu.de (I.H.); mirjam.landgraf@med.lmu.de (M.N.L.); florian.heinen@med.lmu.de (F.H.); 2 LMU Center for Children with Medical Complexity-iSPZ Hauner, LMU University Hospital, LMU Munich, 80337 Munich, Germany; 3 Department of Diagnostic and Interventional Neuroradiology, School of Medicine, Klinikum Rechts der Isar, Technical University of Munich, Ismaninger Str. 22, 81675 Munich, Germany; nico.sollmann@tum.de; 4 TUM-Neuroimaging Center, Klinikum Rechts der Isar, Technical University of Munich, 81675 Munich, Germany; 5 Department of Diagnostic and Interventional Radiology, University Hospital Ulm, Albert-Einstein-Allee 23, 89081 Ulm, Germany

**Keywords:** primary headache, responder rate, neurostimulation, pain pressure threshold, myofascial trigger point

## Abstract

Migraine has a relevant impact on pediatric health. Non-pharmacological modalities for its management are urgently needed. This study assessed the safety, feasibility, acceptance, and efficacy of repetitive neuromuscular magnetic stimulation (rNMS) in pediatric migraine. A total of 13 patients with migraine, ≥6 headache days during baseline, and ≥1 myofascial trigger point in the upper trapezius muscles (UTM) received six rNMS sessions within 3 weeks. Headache frequency, intensity, and medication intake were monitored using headache calendars; headache-related impairment and quality of life were measured using PedMIDAS and KINDL questionnaires. Muscular involvement was assessed using pressure pain thresholds (PPT). Adherence yielded 100%. In 82% of all rNMS sessions, no side effects occurred. All participants would recommend rNMS and would repeat it. Headache frequency, medication intake, and PedMIDAS scores decreased from baseline to follow-up (FU), trending towards statistical significance (*p* = 0.089; *p* = 0.081, *p* = 0.055). A total of 7 patients were classified as responders, with a ≥25% relative reduction in headache frequency. PPT above the UTM significantly increased from pre- to post-assessment, which sustained until FU (*p* = 0.015 and 0.026, respectively). rNMS was safe, feasible, well-accepted, and beneficial on the muscular level. The potential to reduce headache-related symptoms together with PPT changes of the targeted UTM may underscore the interplay of peripheral and central mechanisms conceptualized within the trigemino-cervical complex.

## 1. Introduction

Migraine was one of the most prevalent neurological disorders worldwide in 2019 [1]. In children and adolescents, headache disorders are common and frequently associated with a high burden of disease as well [2,3,4]. Its negative impact on a child’s quality of life, participation in school, sports or leisure time activities, and family life is very high [5,6]. Currently, a multi-modal interdisciplinary approach combining education, lifestyle management, behavioral therapy, and physiotherapy is recommended for children and adolescents affected by migraine [7,8,9,10,11,12,13]. Efficient pharmacological treatments for acute migraine attacks are available, whereas pharmaco-prophylaxis plays a secondary role in pediatric patients due to low evidence levels, oftentimes insufficient efficacy, and the risk of side effects [5,14,15,16]. Whether CGRP antibodies could represent an effective option in the future is currently being evaluated in a randomized clinical trial (https://clinicaltrials.gov/study/NCT03832998 accessed on 20 October 2023). However, data have not yet been published and will only refer to patients affected by chronic migraine. Hence, there is an increasing demand to develop non-pharmacological, non-invasive options as an addition to the contemporary multi-modal approach to pediatric migraine.

Concerning migraine pathophysiology, the trigemino-cervical complex (TCC) plays a major role [17,18,19], which describes the convergence of central and peripheral mechanisms of pain perception, processing, perpetuation, and sensitization [17]. Within this concept, reports of neck pain as well as findings during manual palpation of the neck and upper trapezius muscles (UTMs, e.g., myofascial trigger points (mTrP) [20,21,22,23,24,25,26,27,28,29,30]) can be interpreted as evidence for muscular involvement in patients with migraine [31,32].

The application of repetitive neuromuscular magnetic stimulation (rNMS) targeting the UTMs has been reported to be a safe and well-tolerated treatment option in adults affected by migraine, with encouraging results regarding the decrease in muscular hyperalgesia and headache symptoms [33,34,35]. Similar effects were described in an observational analysis among children and adolescents with headache disorders receiving rNMS in a tertiary outpatient headache center [36,37]. Through painless personalized electromagnetic induction, rNMS provokes an electric current in the stimulated body region [38]. This depolarizes motor and afferent nerves causing, among other effects, the muscle to contract. The resulting increased proprioceptive inflow to the central nervous system is hypothesized to modulate sensorimotor integration and pain processing pathways [15,38,39,40,41,42].

This study was designed to investigate the feasibility of the rNMS intervention in a cohort of children and adolescents affected by migraine by assessing the adherence to, safety of, and satisfaction with the treatment in a prospective design for the first time. In addition, the following clinical endpoints were preliminarily evaluated: changes in headache-related symptoms, including the burden of migraine and in quality of life, as well as the immediate local muscular effects of rNMS in terms of changes in pressure pain thresholds (PPT) above the UTMs.

## 2. Materials and Methods

### 2.1. Ethics and Study Enrollment

This study was approved by the institutional review board (vote 20-194) and registered in the German Clinical Trials Register (DRKS00022141). It was conducted in accordance with the Declaration of Helsinki. Written informed consent was obtained from all participants and their legal guardians.

### 2.2. Subjects

Participants were recruited via the outpatient headache center of our university’s children’s hospital. Inclusion criteria were (1) age 6 to 17 years, (2) a diagnosis of migraine according to the International Classification of Headache Disorders (ICHD 3rd edition) [43], (3) at least six headache days within a 90-day baseline assessment period, and (4) at least one mTrP in one of the UTMs. Regarding mTrP identification, the three standard criteria defining mTrP were carefully checked: (1) a palpable taut band with (2) hypersensitive spots and (3) a referred sensation/pain during manual palpation [22,44]. Exclusion criteria were (1) a diagnosis of familial hemiplegic migraine, (2) any pharmacological migraine prophylaxis except magnesium, (3) any other neurological/psychiatric disorders besides headaches, (4) any serious disease, and (5) contraindications for magnetic stimulation. As mixed-type headache (coexistence of migraine and TTH) is common in children and adolescents, a TTH component was not an exclusion criterion for study participation.

### 2.3. Prospective Study Design and rNMS Intervention

**Figure 1:**
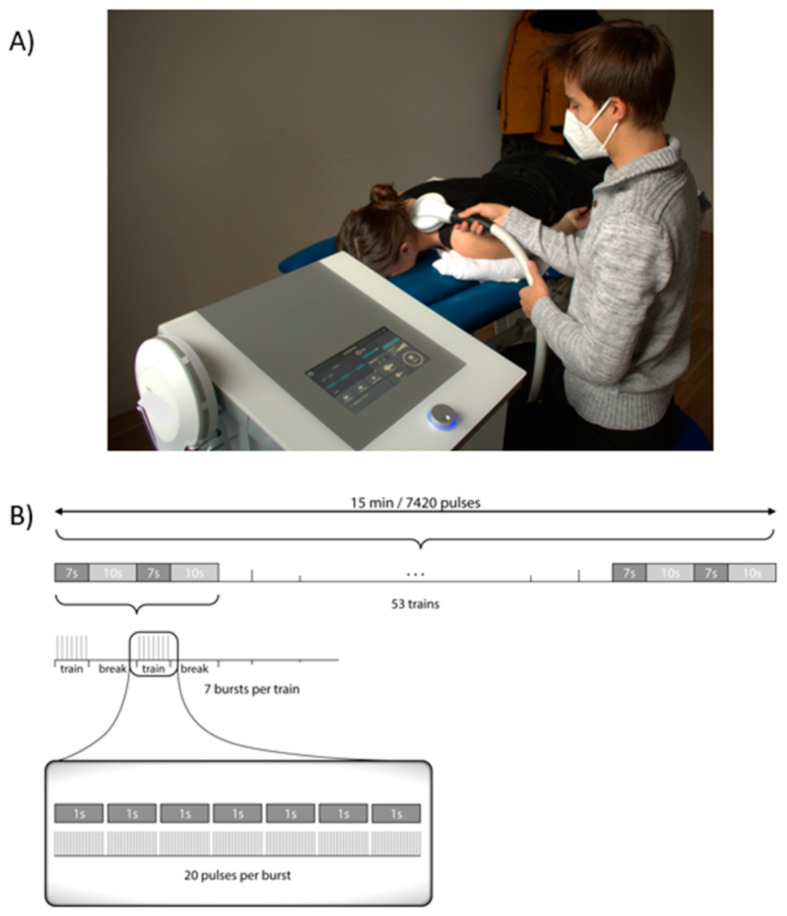
Clinical setup of rNMS treatment. (**A**) rNMS setting and coil positioning. (**B**) Stimulation protocol used for the rNMS treatments. Since 53 trains could not be visualized individually, which is why the repetition of trains is marked with […]. Abbreviation: rNMS = repetitive neuromuscular magnetic stimulation.

Enrollment took place consecutively between August 2020 and October 2021, with the last follow-up examination (FU) taking place in January 2022. During a 90-day baseline period, participants recorded the headache frequency and characteristics using a standardized headache calendar [45]. Subsequently, participants entered a 3-week intervention period consisting of 6 rNMS sessions targeting the UTM bilaterally with an eMFieldPro system (Zimmer Medizinsysteme GmbH, Neu-Ulm, Germany, CE Nr 0123). This study used the rNMS method described in the study of Staisch et al. (2022) and may partly reproduce the wording [36] (15 min, 20 Hz, 7 s ON time, 10 s OFF time; Figure 1). After the intervention, a 90-day FU period took place during in which subjects continued using their headache calendar.

### 2.4. Outcome Measures

This study used similar assessments as described in the study of Staisch et al. (2022) [36] and Börner et al. (2022) [37] and may partly reproduce the wording. Adherence: Adherence was defined as completing at least 5 of the 6 sessions of the rNMS intervention. If sessions were canceled, the reasons were asked for. Safety: A customized standardized questionnaire was used to assess any adverse events (AE) during or after stimulation. Satisfaction: After the intervention, patients and caregivers gave feedback on whether they would like to repeat or recommend rNMS using a customized standardized questionnaire. Clinical outcomes: During the whole course of the study, patients monitored headache symptoms using the headache calendar of the German Migraine and Headache Society [45]. Before the intervention and at FU 90 days after the intervention, headache-related impairment and quality of life were evaluated using the Pediatric Migraine Disability Assessment (PedMIDAS) [46] and a German generic quality of life instrument for patients and caregivers (KINDL questionnaire) [47]. Concurrently, subjects were asked to report life events having occurred during study participation. To identify mTrP in the UTM, a certified physiotherapist examined all participants using manual palpation at the time of screening, before and after the intervention, as well as during the FU exam 90 days after the intervention. In addition to mTrP assessments, reference points were defined as 1/3 and 2/3 of the distance from the vertebra C7 to the acromion above the left and right UTM to allow the investigation of changes in the whole musculature. Before and after each rNMS session as well as at FU examination 90 days after the intervention, PPT above each mTrP and all reference points were determined using algometry (Wagner Instrument, Greenwich, CT, USA). Measurements were performed three times per point.

### 2.5. Data Management

Data were pseudonymized and entered into Microsoft Excel data sheets (Microsoft Office Professional Plus 2016, Microsoft, Redmond, WA, USA). At least two independent analysts checked the data for plausibility. Based on the headache calendars covering 90 days, mean headache frequency, duration, and intensity were reported as headache days per month, hours, and with a 10-point visual analogue scale (VAS) (0 no pain, 10 extreme pain). A headache day was defined as a day with a headache lasting for at least two hours or shorter if headache specific medication was taken (according to the ICHD-3 [43]). Two patients documented the headache intensity on an alternative VAS scale (smaller range) and were therefore excluded from the headache intensity analysis. Two patients noted headache episodes consecutively without the use of the headache calendar template or exact dates, which is why they had to be excluded from the analysis separately comparing headache frequency in the first month, second months, and third months after rNMS treatment as well as from headache intensity and medication intake analyses due to missing information. PedMIDAS scores were available for 12 patients, since 1 patient was at preschool age and therefore not able to complete the PedMIDAS questionnaire as it is partly based on the child’s participation in school. PedMIDAS scores can be categorized as follows: score of 0 to 10: little to none impairment, score of 11 to 30: mild impairment, score of 31 to 50: moderate impairment, and score >50: severe impairment [46]. The maximum pressure of the algometer was 10 kg/cm^2^. If no pain was indicated when reaching 10 kg/cm^2^, this pressure was defined as the PPT [48]. Based on the relative headache frequency reduction from baseline to FU, patients were assigned to one of four responder rate groups (≥75%; ≥50%, ≥25%, <25%) [49]. The FU data regarding PPT were available for 12 patients as one FU examination was only possible via telephone.

### 2.6. Statistics

As this is the first prospective clinical study to deliver rNMS to a pediatric cohort affected by migraine, the study was primarily designed to assess its feasibility in this age group reflected by adherence to the intervention. As the primary endpoint, the adherence rate was calculated as the percentage of participants who did not discontinue the intervention. A threshold of completing at least 5 of the 6 per protocol sessions was defined as fulfilling adherence to the intervention. Assuming that 90% of participants would adhere to the intervention, a sample size of *n* = 12 to *n* = 15 participants was intended to treat based on the expected confidence intervals. For the additional qualitative feasibility endpoints, a sample size estimation was not reasonable. By the time the study had been designed, not any pediatric data for the application of rNMS in migraine were available to base a power analysis with regard to the clinical endpoints on.

All statistical analyses were performed using SPSS (version 26/27; IBM SPSS Statistics for Windows, Armonk, NY, USA). The statistical significance level was set to α = 0.05 for all tests. Adherence rate was defined as the percentage of completed rNMS sessions. Absolute/relative frequencies, means, standard deviations, medians, and ranges were calculated for characteristics, side effects, and the intervention feedback.

Normality of headache variables, questionnaire scores, and PPT were analyzed using Shapiro–Wilk tests. Differences in headache frequency, headache intensity, frequency of days with medication intake, and the KINDL scores of caregivers from baseline to FU were evaluated using paired *t*-tests. Differences in headache duration, PedMIDAS scores, and KINDL scores of participants from baseline to FU were investigated using Wilcoxon signed-rank tests. Differences in monthly headache frequency were compared at 4 time points (baseline, one month, two months, and three months after rNMS treatment, respectively) using a repeated-measures ANOVA. The mean PPT above the left and right UTM was calculated as the average of the PPT above the lateral and medial reference points and the mTrP. Differences in PPT above the left and right UTM were assessed using repeated-measures ANOVAs for the following time points: (1) before the first rNMS session (pre), (2) before the last rNMS session (post), and (3) at FU. For ANOVAs, the Bonferroni correction was used for post hoc comparisons. In the case of a significant Mauchley’s test of sphericity, the Greenhaus–Geisser correction was applied.

## 3. Results

### 3.1. Screening

**Figure 2:**
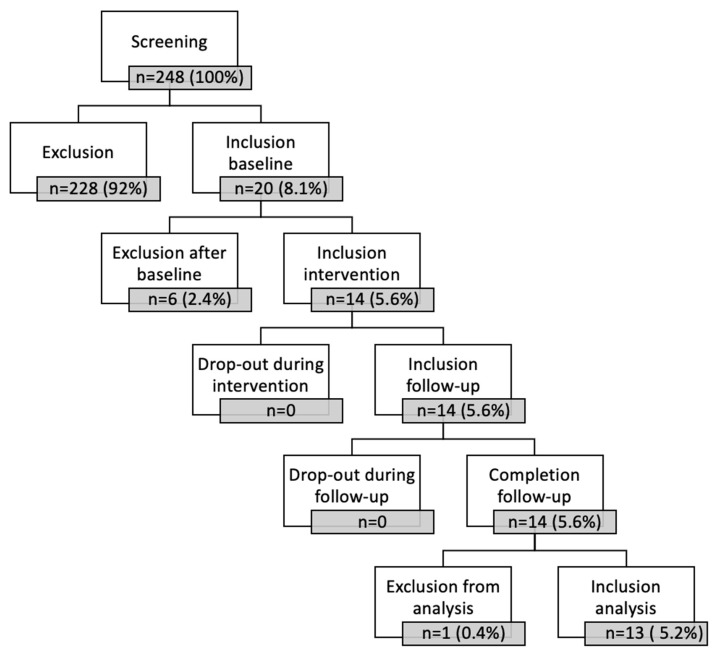
Screening scheme for study inclusion.

A total of 248 patients treated at the outpatient headache center during the enrollment period were screened for eligibility, of whom 20 patients fulfilled all inclusion criteria (8.1%) and completed the baseline period. A total of 6 patients (2.4%) were excluded after the 90-day baseline period due to (1) less than six headache days within the baseline period (*n* = 3), (2) absence of mTrP in the UTM during manual palpation at the end of baseline (*n* = 2), and denial to participate in the intervention period (*n* = 1). One patient was excluded from analysis due to incongruence of the clinical diagnosis and the headache symptoms recorded by the headache calendar (*n* = 1). (Figure 2 and Supplementary Table S1).

### 3.2. Subject Characteristics

A total of 13 patients aged 12.2 ± 3.5 years (range: 6–17 years; 92.3% female) were enrolled in the study (Table 1 and Supplementary Table S2). A total of 3 patients were diagnosed with migraine with aura, of whom 2 patients additionally experienced tension type headache (TTH) characteristics. The remaining 10 patients were diagnosed with migraine without aura, with 5 patients also affected by TTH. The baseline mean headache frequency was 9.43 ± 5.86 headache days per month, with a median of 9.0 and an IQR 4.50–13.17 headache days per month. A total of 7 patients were experiencing neck pain at baseline; 6 patients received physiotherapy during baseline, 2 patients continued, and 1 patient started physiotherapy during the intervention period. All patients took acute medication: most patients used cyclooxygenase inhibitors (*n* = 10 ibuprofen, *n* = 3 naproxen, *n* = 2 acetylsalicylic acid); also triptans (*n* = 4) and paracetamol (*n* = 2) were prescribed. No patient took any preventive migraine medication, except magnesium (*n* = 9). Detailed subject and baseline characteristics are listed in Table 1.

### 3.3. Treatment Characteristics

rNMS was performed with a mean stimulation intensity of 31.8 ± 12.3% of the maximum stimulator output on the left side and at 32.0 ± 11.6% of the maximum stimulator output on the right side.

### 3.4. Adherence

No dropouts were recorded. All participants completed all six rNMS sessions (adherence rate: 100%). Nine patients completed all sessions within a 3-week interval. For four patients, altogether eight sessions needed to be differently scheduled due to (1) acute illness of the patient (*n* = 2, 25%), (2) time constraints by the family (*n* = 1, 12.5%), (3) resource constraints by the outpatient clinic (*n* = 2, 25%), (4) absence without excuse (*n* = 2, 25%), and (5) accident due to weather conditions (*n* = 1, 12.5%) ending up in an intervention period of four to five weeks.

### 3.5. Safety

AEs were evaluated for 78 rNMS sessions. In 64 sessions (82.1%), no AEs were reported. A total of 16 side effects were reported for the remaining 14 rNMS sessions (17.9%) (Table 2). No AE led to discontinuation of the intervention.

### 3.6. Satisfaction

After the intervention, 13 subjects (100%) wanted to repeat rNMS and recommend it to other patients. A total of 13 caregivers (100%) would recommend rNMS to other children with migraine, and 10 caregivers (76.9%) would repeat the intervention. The reason why 3 caregivers did not indicate to repeat the treatment was that they themselves did not perceive a sufficient improvement in their child’s treated headache.

### 3.7. Headache Characteristics 

Headache frequency numerically decreased from 9.43 ± 5.86 days per month during the baseline period by 2.53 days per month to 6.90 ± 4.53 days per month during the FU period. This reduction did not reach statistical significance (t = −1.848, *p* = 0.089, Table 3). Although the numerical drop of the mean monthly headache frequency was pronounced in the first (6.27 ± 4.52 days/month) and second month (6.45 ± 7.12 days/month) compared to the third month (9.00 ± 6.65 days/month) after the intervention, no statistically significant change was reached at any of these timepoints compared to the mean baseline headache frequency (*p* = 0.204, F = 1.76; Supplementary Table S3).

Congruently, we registered a statistically non-significant reduction in medication frequency from 4.42 ± 2.58 days per month at baseline to 2.73 ± 2.10 days per month at FU (t = 1.94, *p* = 0.081) resulting in a mean reduction of 1.7 days per month. Headache intensity and duration did not relevantly change from baseline to FU.

Seven patients were classified as responders showing a relative reduction in headache frequency of ≥25%. Of these seven patients, headache frequency decreased ≥50% in three patients, of which two patients showed a reduction of ≥75%.

### 3.8. Headache-Related Disability 

**Figure 3:**
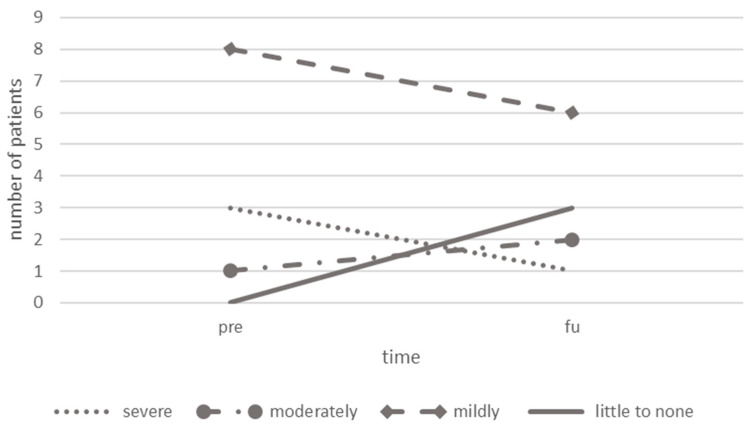
Comparison of PedMIDAS categories before and after rNMS treatment. Abbreviations: rNMS = repetitive neuromuscular magnetic stimulation, pre = before treatment, post = after treatment, PedMIDAS = Pediatric Migraine Disability Assessment.

When comparing PedMIDAS scores at the group-level before intervention (35.00 ± 23.84) and at FU (20.67 ± 16.83), a transition from an average moderate to mild disability was observed (Z = −1.92, *p* = 0.055, Table 3). On the individual level, at baseline, “severe” disability was experienced by three patients, “moderate” disability by one patient, and “mild” disability by eight patients; no patient was categorized as “little to not” disabled (Figure 3). At FU, one patient was categorized as “severely”, two patients as “moderately”, six patients as “mildly”, and three patients as “little to not” disabled. Two patients transitioned to a more severe category, whereas five patients turned to a less severe category, with one patient even dropping from “severe” to “little to none” disability. Five patients remained in their categories. Individual changes from baseline to FU in PedMIDAS scores, monthly headache frequency, intensity, and medication intake are depicted in Supplementary Table S4. No significant change in health-related quality of life was detected, neither in the total score of the KINDL questionnaire answered by the patient (baseline = 65.23 ± 19.02, FU = 67.08 ± 18.04, *p* = 0.675), nor in the questionnaire answered by the caregiver (baseline = 67.27 ± 11.99, FU = 69.44 ± 9.64, *p* = 0.320).

### 3.9. Muscular Effects

**Figure 4:**
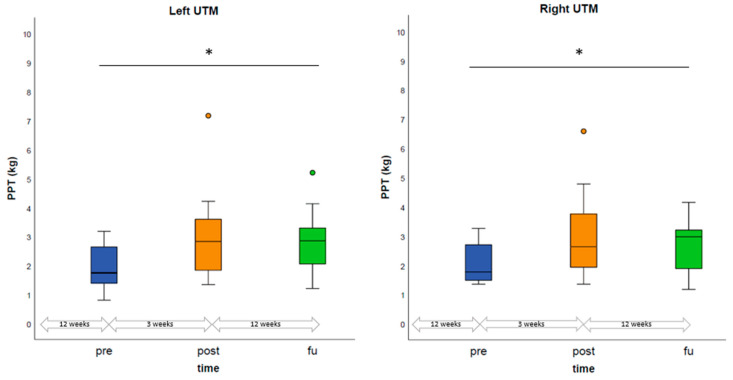
Comparison of PPT prior the first rNMS session, prior the last rNMS session, and at the 3-month FU examination. PPT above the left and right UTM were calculated based on the average of PPT above the lateral and medial reference points as well as the mTrP. Boxplots display the median PPT as well as the IQR. Significant differences are marked with an asterisk (*). Abbreviations: PPT = pressure pain threshold, mTrP = myofascial trigger point, pre = prior to the first rNMS session, post = prior to the last rNMS session, FU = follow-up, IQR = interquartile range.

Mean PPT measured above the left and right UTM significantly increased over time (left UTM: *p* = 0.016, right UTM: *p* = 0.037, Table 4 and Figure 4). Single comparisons of PPT above each assessed point (left lateral, left medial, left mTrP, right lateral, right medial, right mTrP) before and after the rNMS treatment are given in Supplementary Table S5.

Of the seven patients with bilateral mTrP at baseline, one patient had only one unilateral mTrP at FU while the remaining six patients were still diagnosed with bilateral mTrP. Of five patients with unilateral mTrP at baseline (left *n* = one patient, right *n* = four patients), three patients had no mTrP at FU, while mTrP could be detected uni- and bilaterally in one patient, respectively.

## 4. Discussion

This study investigated the feasibility of the rNMS intervention as a non-pharmacological, non-invasive treatment option in a group of children affected by episodic migraine with involvement of the neck muscles. Feasibility measures were the adherence to, safety of, and satisfaction with the treatment. These measures were for the first time assessed in a prospective open-label design in this age group. In addition, preliminary clinical effects of the intervention were prospectively studied for the first time, not only focusing on changes in headache-related and muscular symptoms but on burden of migraine and in quality of life, too.

In this cohort, rNMS was feasible, safe, and well-accepted (adherence rate of 100%; no adverse events in 82.1% of rNMS sessions; 100% of patients would repeat and recommend rNMS). These results are in line with the findings from an observational report of rNMS as treatment in children and adolescents with different types of headache disorders, as well as to the results of studies involving adult participants [34,35,36].

Regarding the effects on headache-related symptoms, the monthly headache frequency and medication intake numerically decreased after the intervention, albeit without statistical significance. Importantly, seven patients (54%) were qualified as responders by experiencing a relief of their headache frequency by at least 25% and one additional participant reported a reduction close to the responder threshold (23%). This trend is comparable to findings in a cohort of children and adolescents with different types of headache disorders receiving rNMS, in that headache frequency and intensity were significantly reduced after rNMS (reduction from 17.1 ± 11.4 to 10.9 ± 10.9 headache days/month [mean ± SD]) [36] and comparable responder rates were observed for the group of participants affected by primary headaches, including nine patients with mixed-type headache and two with migraine only (43% responders in terms of ≥25% reduction, 14% responders in terms of ≥75% reduction) [36,37]. Similar findings have also been reported in previous studies investigating rNMS in young adults with episodic migraine (reduction in headache frequency from 7.7 (5.7–12) to 5.3 (1.7–10.3) days/month [median (range)] and 7.7 ± 6.9 to 5.1 ± 4.8 days/month [mean ± SD]; reduction in medication intake from 4 (0–9.7) to 3 (0–9) days/month [median (range)] and 3.3 ± 2.8 to 2.8 ± 1.8 days/month [mean ± SD]) [33,35]. Moreover, a retrospective analysis of the pooled data of both studies showed similar developments, too (reduction in headache frequency from 8.17 ± 4.50 to 6.33 ± 4.38 days/month [mean ± SD], reduction in medication intake from 3.63 ± 2.58 to 3.10 ± 2.44 days/month [mean ± SD]) [50].

With regard to headache-related disability, a significant reduction in MIDAS scores was reported after rNMS in adults with episodic migraine in previous studies (MIDAS Score reduction from 26.33 ± 13.89 to 15.37 ± 12.30 [mean ± SD]) [50]. In congruence, in the current first ever report on the impact of rNMS to the burden of migraine, PedMIDAS scores decreased on average by 14.33 units from baseline to FU, which corresponds to a reduction of 40.9%. These results are clinically meaningful, considering the highly problematic consequences of school absenteeism, and avoidance of physical and everyday activities in childhood due to migraine symptoms. Thus, a decreased PedMIDAS score likely reflects increased participation after rNMS, representing an important criterion regarding the treatment of pediatric migraine. In our pediatric cohort, more patients were classified as being mildly to not at all disabled after the rNMS intervention. With regard to the KINDL scores, no changes in health-related quality of life were reported after rNMS, neither by patients nor by their caregivers. However, it should be noted that baseline KINDL scores (65.23 ± 19.02) were already almost at the same level as reference values of healthy children in the KIGGS study (“Studie zur Gesundheit von Kindern und Jugendlichen in Deutschland”, performed by the Robert Koch-Institute; mean: 76.90; 95% confidence interval 76.70–77.10) [51] and BELLA study (“BEfragung zum seeLischen WohLbefinden und VerhAlten”, submodule of the KIGGS study; 76.30 ± 10.10) [52]. Hence, ceiling effects may have hampered the detection of further improvement.

Regarding muscular effects, the current analysis demonstrated an increase in PPT from pre- to post-assessments above the UTM, reflecting a relief of muscular hypersensitivity. This effect lasted until the 3-month FU, implicating that a decrease in local muscular hypersensitivity induced by rNMS can be sustained for a certain period of time. Of importance, in contrast to the long-term muscular effects of rNMS portrayed in this study, the majority of studies evaluating the effects of neuromodulation methods only included acute short-time FU (e.g., FU period of 10 min) [53,54,55,56,57,58,59,60]. Our findings are congruent with previous investigations of rNMS in children [37] as well as in adults affected by headaches. Our results show an increase in PPT from pre- to post-assessment of 0.96 ± 0.42 kg/cm^2^ for the right and 1.03 ± 0.42 kg/cm^2^ for the left UTM, which is comparable to PPT increases reported in the adult studies (right UTM: 0.4 (−1.1–2.5) kg/cm^2^ [median (range)] and 0.8 kg/cm^2^ [mean, SD for difference not given]; left UTM: 0.6 (−0.5–2.6) kg/cm^2^ [median (range)] and 0.6 kg/cm^2^ [mean, SD for difference not given]) [34,35]. PPT after rNMS measured above the UTM increased to a level of PPT measured in pediatric patients with chronic pain above the non-pain control sites and in a healthy reference population [61]. In addition, PPT above the UTM prior to rNMS were comparable or lower than PPT measured in adult migraine patients. PPT after rNMS were similar or even higher than PPT of healthy controls [62,63]. Together, this suggests an even more pronounced muscular hypersensitivity in pediatric patients than in healthy adults, which can potentially be reset to a level of healthy controls by rNMS targeting the UTM as a muscle considered part of the TCC. This may be interpreted as a sign of network reorganization via the TTC, eventually including the desensitization of the hypersensitive trigeminal nucleus caudalis [64].

Regarding the aspect of neuroinflammation in migraine pathogenesis, magnetic resonance imaging (MRI) studies suggest neuroinflammatory mechanisms on the muscular level [65,66], in addition to the well-described CGRP-related (Calcitonin Gene-Related Peptide) alterations on the leptomeningeal vascular level [67,68]. The relief of muscular symptoms (e.g., increased PPT, decreased number of mTrP) by rNMS points at a possible relief from muscular neuroinflammation. In addition to the beneficial clinical effects, the important interplay of the peripheral and central networks is emphasized, once more. This context might call for further in-depth investigations of alterations of muscles involved in migraine pathogenesis via the TCC, i.e., by advanced imaging on behalf of T2 mapping and other advances MRI-based techniques [65,66].

Neurostimulation as acute or prophylactic migraine treatment is quite a novel approach; thus, the number of studies is still limited to date and no data exist for the pediatric population for the majority of modalities [7,69]. For the acute migraine treatment and migraine prophylaxis, the following approaches have been investigated: transcranial magnetic stimulation [70], transcranial direct current stimulation [71], transcutaneous occipital nerve stimulation [53,72], transcutaneous supraorbital nerve stimulation [54,73], transcutaneous vagus nerve stimulation [74,75], and remote electrical neuromodulation [60]. In comparison to the abovementioned techniques, rNMS specifically targets the muscle and could treat the muscular level in addition to central effectors—including in children and adolescents. Specifically, it has several aspects that might facilitate its use in the pediatric setting, including in particular a painless application. Therefore, rNMS may be better accepted by patients, which is an important factor in the pediatric field [36,69,76]. Regarding the association of migraine, neck pain, and muscular hypersensitivity, rNMS is unique in targeting both the muscular and the central pathophysiological mechanisms conceptualized within the framework of the TCC [17,19], which is achieved via a single “from bottom-up” approach [69]. Thus, rNMS may represent a valuable non-invasive, non-pharmacological component within the future treatment concepts for pediatric migraine. Against the background of these promising results in children, data from large-scale randomized controlled trials in adults are expected to pave the way for a widespread application of rNMS across all age groups (https://drks.de/search/de/trial/DRKS00024470 accessed on 20 October 2023).

As this study included a rather small cohort of 13 patients with migraine, findings are not generalizable to the whole population of pediatric patients affected by migraine. Despite the small cohort, the assumption that 90% of participants would adhere to the intervention led to a sample size calculation of *n* = 12 to *n* = 15 participants needing to be treated to reach reasonable confidence intervals (CI ± 15.2 to ± 16.9), which enhances the reliability of the effects despite the sample size limitation. Yet, given the strict in- and exclusion criteria, the data represent the feasibility and preliminary effects in a cohort of pediatric patients affected by migraine as clinically homogenously as possible. In particular, assuring a relatively high baseline frequency of headaches and the presence of muscular involvement through an expert manual palpation, together with the rule out of comorbidities like somatoform or psychiatric disorders, represent important quality criteria of the study. Another reason for limitations in the sample size had been the ongoing COVID-19 pandemic, that restricted outpatient contacts to a minimum. Given the age range of the study population, no conclusions regarding when to start a neurostimulation during the trajectory of migraine can be drawn. Headache documentation is especially challenging in children and adolescents, which should be considered when interpreting the reported results. Novel, digitalized kids-friendly applications are urgently needed to ensure a more feasible headache documentation in clinical practice and research. Regarding muscular effects, only one FU examination took place 90 days after the intervention. While numerically decreased headache frequency was pronounced during the first and second month after the intervention, no conclusions regarding trajectory or wear-off effects regarding the muscular symptoms can be made to date. Future studies should therefore consider implementing physiotherapeutic assessment at several time points during FU and may additionally implement objective point-of-care imaging measures to assess muscular changes (e.g., muscular ultrasound or infrared thermography). Furthermore, the lack of objective neurophysiological outcome measures (e.g., fMRI) limits the interpretability of the here-reported rNMS effects, and further studies including neurophysiological outcome measures are needed to underpin the pathophysiological hypothesis of the distinct mechanisms of action of rNMS in migraine. Concerning algometry, it should be noticed that measurements in young children (6–8 years) may not be as reliable as in adults or older children, which is due to difficulties in describing perceptions and a higher sensitivity to pain stimuli [63]. A setting effect may have affected the here-presented outcomes, especially since this effect might be higher in the pediatric population in general [5,11,77]. Furthermore, there may be an increased placebo response to interventions using a medical device compared to pharmacologic treatment modalities [77]. In addition, three psychosocial AEs were reported by three patients during the study period, which may have interfered with the effects reported here. Since the study was carried out during the COVID-19 pandemic, the closure of schools, sports clubs, and recreational facilities, social distancing, and the rapid change in legal restrictions may have affected the patients’ daily routine, as well as overall quality of life and burden of headache. Since migraine is a very common disorder in pediatric age but nevertheless characterized as one of the most underfunded diseases [78,79], more sham-controlled studies investigating non-pharmacological, non-invasive treatment options for pediatric patients are urgently needed.

## 5. Conclusions

rNMS interventions were safe, feasible, and well-accepted by children and adolescents with migraine. Although statistically non-significant, the monthly headache frequency, medication intake, and—particularly important and reported for the first time in this context—PedMIDAS scores demonstrated a relevant decrease from baseline to FU on an individual basis. Together with the potential to reduce the symptoms on the muscular level, rNMS might become a valuable option introducing neuromodulation from bottom up to the multimodal armentarium for children with episodic migraine. Therefore, future controlled studies are highly needed to further assess the current beneficial findings and to elucidate the specific neurophysiological mechanisms of rNMS in peripheral and central processes of pain processing.

## Figures and Tables

**Table 1:** Characteristics of study participants (*n* = 13).

Characteristics		*n* (%)	Median (Range)
Age		-	12 (6–17)
Sex	Female	12 (92.3%)	-
Handedness	Right	10 (76.9%)	-
Headache Diagnosis			
Migraine with aura		1 (7.7%)	-
Migraine without aura		5 (38.5%)	-
Migraine with aura + TTH		2 (15.4%)	-
Migraine without aura + TTH		5 (38.5%)	-
Age at headache onset (years)		-	9 (2–15)
Time since headache onset (years)		-	3 (2–13)
Family history for migraine	Yes	3 (23.1%)	-
No		9 (69.2%)	-
Not known		1 (7.7%)	-
Neck pain at baseline	Yes	7 (53.8%)	-
No		6 (46.6%)	-
mTrP localization at baseline	Unilateral	5 (38.5%)	-
	Bilateral	8 (61.5%)	-
	Left	10 (45.5%)	-
Right		12 (54.5%)	-
mTrP entity at baseline	Latent	15 (68.2%)	-
Active		7 (31.8%)	-
Physiotherapy	During baseline	6 (46.2%)	-
During intervention		3 (23.1%)	-

Abbreviations: TTH = tension type headache, mTrP = myofascial trigger point.

**Table 2:** Adverse events (AEs) documented within *n* = 78 rNMS sessions.

AE (*n* = 91)	*n* (%)	Serious/Severe	Unexpected	Related
No side effects	64 (82.1%)			
Side effects	16 in 14 sessions (17.9%)			
During stimulation				
Trembling (arm/hand)	5 (6.4%)			X
Heaviness (at stimulation site)	2 (2.6%)			X
Tingling (at stimulation site)	1 (1.3%)			X
Arm pain	1 (1.3%)			X
Tension in shoulder-neck region (hand)	1 (1.3%)			X
In-between stimulations				
Headache	5 (6.4%)			X
Sore muscles	1 (1.3%)			X
Life events				
Suicide of school colleague	1 (7.7%)	X	X	
Health-related absence of caregiver ^a^	1 (7.7%)			
Accident on ice	1 (7.7%)		X	

^a^ For this variable, none of the criteria (serious/severe, unexpected, related) applied; they might have influenced the perception of headaches. Abbreviation: AE = adverse event.

**Table 3:** Change in headache characteristics, PedMIDAS scores, and KINDL scores from baseline to FU.

Headache Characteristics	Pre		FU		Test Values		95% CI of Mean Difference
	Mean (SD)	Median (IQR)	Mean (SD)	Median (IQR)	*t*/*Z*	*p*	
Headache frequency	9.43 (5.86)	9.00 (4.50–13.17)	6.90 (4.53)	5.60 (3.00–10.67)	*t* = 1.848	0.089	−0.45–5.52
Headache intensity	5.50 (0.97)	5.21 (4.75–6.73)	6.27 (1.47)	6.53 (4.24–7.09)	*t* = −1.68	0.142	−1.86–0.31
Headache duration	6.27 (4.82)	5.03 (3.56–7.35)	6.50 (4.70)	4.45 (2.59–9.41)	*Z* = −0.89	0.929	-
Medication frequency	4.42 (2.58)	4.33 (2.67–5.33)	2.73 (2.10)	2.00 (0.75–4.66)	*t* = 1.94	0.081	−0.25–3.65
PedMIDAS	35.00 (23.84)	24.00 (21.00–51.00)	20.67 (16.83)	16.00 (7.75–30.75)	*Z* = −1.92	0.055	-
KINDL Child	65.23 (19.02)	69.50 (46.13–82.75)	67.08 (18.04)	74.00 (58.38–79.25)	*Z* = −0.420	0.675	-
KINDL Caregiver	67.27 (11.99)	68.75 (58.38–77.63)	69.44 (9.64)	70.75 (61.50–78.75)	*t* = −1.038	0.320	−6.74–2.39

Comparisons were made using paired-samples *t*-tests or Wilcoxon signed-rank tests depending on normality. Abbreviations: pre = before the rNMS intervention, FU = follow-up, SD = standard deviation, IQR = interquartile range, CI = confidence interval, KINDL = Revidierter Fragebogen für KINDer und Jugendliche zur Erfassung der gesundheitsbezogenen Lebensqualität, PedMIDAS = Pediatric Migraine Disability Assessment.

**Table 4:** PPT comparison above the left and right UTM prior the first rNMS session (pre), prior the last rNMS session (post), and at the 3-month FU examination.

	Test Values			Mean_Pre (SD)	Mean_Post (SD)	Mean_FU (SD)	Post Hoc Test
	*F*	*p*	*η* ^2^				*p*
Left UTM	6.46	0.016 *	0.564	1.99 (0.77)	3.02 (1.61)	2.84 (1.13)	
Pre-post							0.097
Pre-FU							0.015 *
Post-FU							1.000
Right UTM	4.67	0.037 *	0.483	2.04 (0.67)	3.00 (1.55)	2.70 (1.00)	
Pre-post							0.126
Pre-FU							0.026 *
Post-FU							1.000

PPT comparisons above the left and right UTM using repeated-measures ANOVAs. Post hoc comparisons were performed with Bonferroni correction. Significant differences at α = 0.05 are marked with an asterisk (*). Abbreviations: PPT = pressure pain threshold, UTM = upper trapezius muscle, FU = follow-up, pre = prior the first rNMS session, post = prior the last rNMS session, SD = standard deviation.

## Data Availability

The data presented in this study are available on request from the corresponding author. The data are not publicly available due to the sensitive character of pediatric clinical data.

## Supplementary Materials

The following supporting information can be downloaded at: https://www.mdpi.com/article/10.3390/children10111764/s1; Table S1: Screening. Screening and reasons of exclusion; Table S2: Participants’ characteristics. More detailed characteristics of the study participants; Table S3: Changes in monthly headache frequency of *n* = 11 patients for the first month, second month, and third month after rNMS intervention compared to baseline; Table S4: Individual changes from baseline to FU in PedMIDAS scores, monthly headache frequency, headache intensity, and medication intake; Table S5: (A) Comparison of PPT before the first (pre1) and before the last treatment sessions (pre6). (B) Comparison of PPT after the first (post1) and after the last treatment sessions (post6).
