# Supplementary material for: Neuromodulation in Pediatric Migraine using Repetitive Neuromuscular Magnetic Stimulation: A Feasibility Study
Source: Children (Basel). 2023 Oct 30;10(11):1764. doi: 10.3390/children10111764 (PMC10670480; doi:10.3390/children10111764)
Supplement: Supplementary file 1 [file children-10-01764-s001.zip › children-2659846-supplementary.pdf]

Supplements

Supplementary Table S1: Screening. Screening and reasons of exclusion.

Reason of ineligibility	n	%
Screening	248	100
Exclusion	228	92
No migraine diagnosis	84	36.9
No mTrP in M. trapezius	57	25
Not in age range (< 6 years, > 18 years)	13	5.7
Headache frequency not applicable (< 6 days in 3 months)	32	14
Pharmacological prophylaxis	3	1.3
Familial hemiplegic migraine	2	0.9
Neurological/psychiatric disorder	15	6.6
Other severe disease	4	1.8
Epilepsy	1	0.4
No consent: no interest	10	4.4
No consent: too time-consuming	6	2.6

No consent: distance b/w home and treatment too far	1	0.4
**Exclusion after baseline**	6	2.4
Headache frequency not applicable (< 6 days in 3 months)	3	50
mTrP after baseline not applicable	2	33.3
No consent: no interest	1	16.7
**Inclusion**	14	5.6
**Exclusion from analysis**	1	0.4

Abbreviations: mTrP = myofascial trigger point

Supplementary Table S2: Participants' characteristics. More detailed characteristics of the study participants.

Characteristics	n(%)	Characteristics	n(%)
Other diagnoses (multiple answers possible)		Headache characteristics (multiple answers possible)	
Cyclic vomiting	2 (15.4%)	Pulsating	8 (61.5%)
Scoliosis	1 (7.7%)	Dull/pressing	9 (69.2%)
Tricuspid insufficiency	1 (7.7%)	Pulling	2 (15.4%)
Underweight	1 (7.7%)	Not known	2 (15.4%)
Vitamin D deficiency	1 (7.7%)	Vomiting	5 (38.5%)
Myopia	1 (7.7%)	Nausea	11 (84.6%)
Hyperopia	1 (7.7%)	Photophobia	10 (76.9%)
Sleep disturbances	1 (7.7%)	Phonophobia	11 (84.6%)
Asthma	1 (7.7%)	Odor-sensitive	2 (15.4%)
Allergies	1 (7.7%)	Tearing eyes	1 (7.7%)
Flat foot	1 (7.7%)	Running nose	1 (7.7%)
Headache triggers (multiple answers possible)		Visual disturbances	2 (15.4%)

No triggers	5 (38.5%%)	Acute medication (multiple answers possible)	
Stress	3 (23.1%)	No medication	
Regeneration	1 (7.7%)	Ibuprofen	10 (76.9%)
Heat	1 (7.7%)	Naproxen	3 (23.1%)
Media	1 (7.7%)	Triptan	4 (30.8%)
Wheat	1 (7.7%)	Paracetamol	2 (15.4%)
Noise	1 (7.7%)	Acetylsalicylic acid (ASS)	2 (15.4%)
Car	1 (7.7%)	Magnesium prophylaxis	
Bicycle	1 (7.7%)	Yes	9 (69.2%)
Prodromal symptoms		No	4 (30.8%)
No precursors	8 (61.5%)		
Flicker	4 (30.7%)		
Dizziness	1 (7.7%)		
Headache localization			
Bilateral	7 (53.8%)		
Unilateral	2 (15.4%)		
Mixed	4 (30.8%)		

Supplementary Table S3: Changes in monthly headache frequency of n=11 patients for the first month, second month, and third month after rNMS intervention compared to baseline.

**Time point**	**Monthly headache frequency** Mean (SD)	***F***	***p***
		1.76	0.204
**Baseline**	10.03 (6.20)		
**FU 1**	6.27 (4.52)		
**FU 2**	6.45 (7.12)		
**FU 3**	9.00 (6.65)		

Pairwise comparisons showed no significant differences between time points. Abbreviations: SD = standard deviation, FU 1 = follow-up one month after rNMS, FU 2 = follow-up two months after rNMS, FU 3 = follow-up three months after rNMS.

Supplementary Table S4: Individual changes from baseline to FU in PedMIDAS scores, monthly headache frequency, headache intensity, and medication intake.

**Patient Number**	**Individual changes from baseline to FU (%)**			
	PedMIDAS score	Headache frequency in days/month	Headache intensity in VAS	Medication intake in days/month
1	-2.00 (-10)	1.34 (31)	-0.99 (-19)	1.00 (23)
2	-13.00 (-93)	-2.00 (-100)	-	-
3	-5.00 (-24)	6.70 (74)	-0.35 (-5)	0.33 (7)
4	-11.00 (-46)	-5.00 (-38)	-1.11 (-23)	-2.00 (-67)
5	10.00 (21)	0.67 (13)	-	-
6	-9.00 (-60)	-3.66 (-50)	0.59 (9)	-3.00 (-60)
7	-92.00 (-99)	-11.33 (-79)	-0.73 (-11)	-8.67 (-81)
9	-22.00 (-42)	-3.00 (-23)	1.37 (31)	-0.66 (-50)
10	20.00 (95)	-0.67 (-6)	1.75 (28)	-0.01 (-0.4)
11	-10.00 (-38)	-1.66 (-36)	2.52 (61)	-1.00 (-33)
12	-30.00 (-49)	-1.33 (-11)	1.53 (30)	0.34 (5)
13	0 (0)	-12.00 (-52)	3.15 (61)	-5.00 (-94)
14	-8.00 (-33)	-1.00 (-30)	-	0 (0)

Abbreviations: FU = follow-up

Supplementary Table S5. A) Comparison of PPT before the first (pre1) and before the last treatment sessions (pre6). B) Comparison of PPT after the first (post1) and after the last treatment sessions (post6).

	**A) Pre1-pre6 comparison**						**B) Post1-post6 comparison**					
	**Test values**		**Mean pre (SD)**	**Mean post (SD)**	**Confidence interval**		**Test values**		**Mean pre (SD)**	**Mean post (SD)**	**Confidence interval**	
**PPT**	***t/Z***	***p***			Lower value	Upper value	***t/Z***	***p***			Lower value	Upper value
Left lateral	*t* = -3.06	.010*	1.83 (.86)	2.91 (1.54)	-1.85	-.31	*t* = -2.56	.025*	2.09 (.84)	3.27 (2.00)	-2.18	-.17
Left medial	*Z* = -1.99	.046*	2.04 (.81)	2.83 (1.67)	-	-	*t* = -2.22	.047*	2.16 (.93)	3.23 (1.86)	-2.11	-.02
Right medial	*t* = -2.02	.066	2.02 (.94)	2.90 (1.61)	-1.84	.07	Z = -1.99	.046*	1.98 (.79)	2.99 (1.67)	-	-
Right lateral	*t* = -3.00	.011*	1.86 (1.09)	2.80 (1.39)	-1.62	-.26	*t* = -2.91	.013*	1.97 (1.12)	3.31 (2.02)	-2.33	-.34
TrP left	*t* = -2.02	.078	1.77 (.57)	3.01 (1.96)	-2.67	.02	*t* = -2.29	.052	2.03 (.72)	3.52 (2.33)	-3.00	-.23

TrP right	*t* = -2.79	.019*	1.66 (.71)	3.10 (1.85)	-2.59	-.29	*t* = -2.00	.073	2.04 (.73)	3.18 (2.18)	-2.41	.13

Comparisons of PPT using paired t-tests or Wilcoxon tests depending on normality. Significant differences at α=0.05 are marked with an asterisk (*).
Abbreviations: PPT = pressure pain threshold, SD = standard deviation
